# Human physiologically based pharmacokinetic model for propofol

**DOI:** 10.1186/1471-2253-5-4

**Published:** 2005-04-22

**Authors:** David G Levitt, Thomas W Schnider

**Affiliations:** 1 Department of Physiology, University of Minnesota, 6–125 Jackson Hall, 321 Church St. S. E., Minneapolis, MN 55455, USA; 2 Institut für Anästhesiologie, Kantonspital, CH-9007 Saint Gallen, Switzerland

## Abstract

**Background:**

Propofol is widely used for both short-term anesthesia and long-term sedation. It has unusual pharmacokinetics because of its high lipid solubility. The standard approach to describing the pharmacokinetics is by a multi-compartmental model. This paper presents the first detailed human physiologically based pharmacokinetic (PBPK) model for propofol.

**Methods:**

PKQuest, a freely distributed software routine , was used for all the calculations. The "standard human" PBPK parameters developed in previous applications is used. It is assumed that the blood and tissue binding is determined by simple partition into the tissue lipid, which is characterized by two previously determined set of parameters: 1) the value of the propofol oil/water partition coefficient; 2) the lipid fraction in the blood and tissues. The model was fit to the individual experimental data of Schnider et. al., Anesthesiology, 1998; 88:1170 in which an initial bolus dose was followed 60 minutes later by a one hour constant infusion.

**Results:**

The PBPK model provides a good description of the experimental data over a large range of input dosage, subject age and fat fraction. Only one adjustable parameter (the liver clearance) is required to describe the constant infusion phase for each individual subject. In order to fit the bolus injection phase, for 10 or the 24 subjects it was necessary to assume that a fraction of the bolus dose was sequestered and then slowly released from the lungs (characterized by two additional parameters). The average weighted residual error (WRE) of the PBPK model fit to the both the bolus and infusion phases was 15%; similar to the WRE for just the constant infusion phase obtained by Schnider et. al. using a 6-parameter NONMEM compartmental model.

**Conclusion:**

A PBPK model using standard human parameters and a simple description of tissue binding provides a good description of human propofol kinetics. The major advantage of a PBPK model is that it can be used to predict the changes in kinetics produced by variations in physiological parameters. As one example, the model simulation of the changes in pharmacokinetics for morbidly obese subjects is discussed.

## Background

Propofol is widely used for the induction and maintenance of anesthesia and as a sedative in intensive care units where it is given as a constant intravenous infusion for periods of many days. In addition to its clinical importance, propofol provides a valuable model for understanding the human pharmacokinetics of agents that are concentrated in fat. Propofol has an oil/water partition coefficient (K_oil_) of about 4700 [1], one of the largest of any pharmacological agent. In comparison, the highly lipophilic volatile anesthetics, such as halothane, have a K_oil _of less than 300 [2]. Because of this large fat partition, propofol is highly concentrated in adipose tissue where it has slow uptake and release kinetics.

This paper presents the first detailed physiologically based pharmacokinetic (PBPK) description of human propofol pharmacokinetics. The model describes the pharmacokinetics in terms of realistic human parameters, such as the organ blood flow and the tissue/blood partition. The PBPK model is implemented in PKQuest, a new software routine that has now been applied to more than 25 different solutes with a wide range of pharmacokinetic properties [3-9].

The major limitation in most human PBPK models is the uncertainty in the values used for the tissue/blood partition coefficients, which cannot be directly measured and are usually based on uncertain extrapolations from animal measurements. In general, the tissue partition of solutes has a complex dependence on protein and lipid binding and can vary markedly from tissue to tissue [10-13]. This means that the PBPK model is dependent on a large number of individual tissue partition coefficients that are not well characterized and, effectively, become adjustable model parameters. However, the highly fat soluble non-polar solutes, such as the volatile anesthetics, are a special case. Their tissue/blood partition is dominated by simple, non-specific partition into the tissue lipid. In a previous application of PKQuest to the volatile anesthetics [4], it was shown that the water/tissue partition could be directly determined just from a knowledge of the fraction of lipid in the different tissues and the value of lipid/water partition coefficient (K_oil_). This means that once the tissue lipid fractions are known (which are not solute dependent), the tissue/blood partition coefficient for any solute of this type is completely characterized by knowledge of the just the one physical parameter, the K_oil_. The PBPK modeling of solutes of this type is not only greatly simplified, but one can have more confidence in the model predictions because of the elimination of most of the adjustable parameters.

It is assumed here that this same approach can be applied to propofol. The tissue/blood partition coefficient is equal to the ratio of the tissue/water and blood/water partition:



The tissue/water partition is determined from the fraction of lipid in the tissue and the K_oil _of propofol. The blood/water partition is determined from experimental measurements of the fraction of propofol that is free (unbound) in blood – defined by the parameter freepl.. Since there is large individual variation in the value of freepl [14-19] one might regard it as an adjustable parameter that varied from subject to subject. However, it was found that the PBPK model adequately predicted the individual results using one average value of freepl for all subjects. Only one parameter was adjusted for each subject – the intrinsic liver clearance. All the other PBPK parameters are identical to those that have been used previously in the application of PKQuest to a variety of solutes. A detailed description of the propofol PBPK model is provided in the Methods Section.

This PBPK model is evaluated by applying it to the experimental data of Schnider et. al. [20]. This data describes the arterial pharmacokinetics of propofol in 24 healthy volunteers with ages varying from 24–81 years, and at 4 different doses. Each subject was given an initial bolus dose, followed 60 minutes later by a constant 60-minute infusion. In the original publication, the pharmacokinetics for the constant infusion phase was interpreted in terms of a 6 parameter compartmental model using NONMEM. Surprisingly, the kinetic parameters from the constant infusion phase were poor predictors of the kinetics following the bolus injection in the same subject. This suggested that there was some systematic difference between the bolus and infusion kinetics, and effects such as early recirculation, a propofol induced change in liver blood flow or pulmonary sequestration were listed as possible explanations. This new PBPK analysis suggests that pulmonary sequestration is the major factor responsible for this discrepancy. The propofol is formulated as a lipid emulsion because it has a low aqueous solubility. In some subjects, a significant fraction of the bolus emulsion (0 – 60%) is sequestered in its first pass through the lung, and then slowly released. When a quantitative model (with two additional adjustable parameters) of this sequestration is incorporated into the PBPK model, a single set of PBPK parameters provides a good description of both the bolus and constant infusion phases.

## Methods

### Experimental data

The methodology for the data collection and analysis was described in detail in the previous publication of Schnider et. al. [20]. Briefly, 24 volunteers in 3 age groups (18–34; 35–65; and >65 years) of 8 each were selected. The individual subjects will be identified as, eg, Subject # 1–5 where the first number refers to the age group (1: 18–34 years, etc.) and the second number is the individual number for that group. Each subject was given an initial bolus (≈ 20 second) dose (2 mg/kg for subjects < 65; 1 mg/kg for subjects >65) followed 60 minutes later by a constant 60 minute infusion of 25, 50, 100 or 200 μg/kg/min. Arterial blood samples were taken at 0, 1, 2, 4, 8, 16, 30, 60, 62, 64, 68, 76, 90, 120, 122, 124, 136, 150, 180, 240, 300 and 600 minutes and the arterial plasma propofol concentration was determined. Each subject was studied on two separate visits, using either propofol with or without EDTA under otherwise identical infusion conditions. Since there was no significant difference with or without EDTA, the data from these two experiments were averaged and were used as the individual data that was fitted with the PBPK model.

The percent body fat for each subject was determined using the regression equation of Gallagher et. al. [21] with an additional correction for Asians [22]:



where Sex is 0 for females and 1 for males, Asian is 1 for Asians and 0 for others, Age is in years and BMI = weight/height^2 ^(kg/m^2^). Although the only value that is directly used in the PBPK calculation for each subject is the percent fat, the age, weight, sex, height and ethnicity enter as covariate parameters through eq. (2).

### Description of the PBPK tissue model for propofol

**Figure 1:**
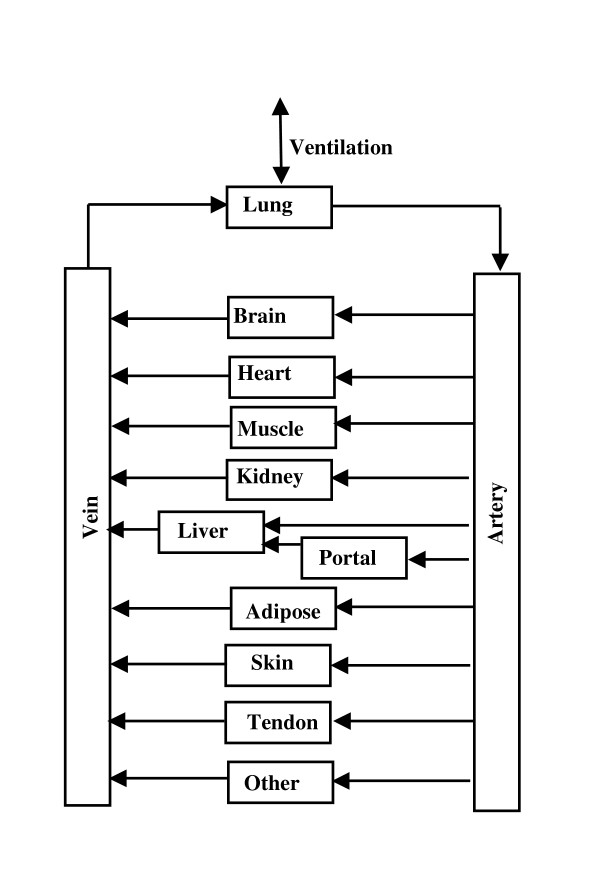
Schematic diagram of the PBPK model. The organ "portal" refers to all the organs drained by the portal vein. The connective tissue is divided between two organs: "tendon" with a relatively low blood flow and "other" with a higher blood flow.

The PBPK model is identical to the one that has been used in previous applications of PKQuest (fig. 1). The model parameters (organ blood flow, volume, etc.) are identical to those used in previous applications of PKQuest [3-9]. The connective tissue is divided between two organs: "tendon" with a relatively low blood flow, and "other" with a higher blood flow [8]. The PBPK values for the tissue weight, blood flow and fraction lipid are listed in Table 1. A major limitation of this PBPK analysis is that a constant set of "standard human" resting organ blood flow is assumed and any hemodynamic changes associated with the uptake and washout of propofol are ignored. The experimental observation that cardiac output is either unchanged [23,24] or only slightly decreased [25,26] with short term propofol supports this assumption. However, Sellgren et. al. [25,26] reported that propofol produced large changes in peripheral blood flow which would suggest that there may be a significant redistribution in the organ blood flow that is ignored in this PBPK model.

**Table 1:** Standard human PBPK organ weights, blood flows and fraction lipid

**Organ**	**Weight (Kg)**	**Fraction Lipid**	**Perfusion (L/Kg)**	**Total Flow (L/min)**
**Blood**	5.5	≈0.01		
**liver**	1.8	0.25	0.25	0.45
**portal**	1.5	0.02	0.75	1.125
**muscle**	26	0.017	0.0225	0.585
**kidney**	0.31	0.017	4	1.24
**brain**	1.4	0.022	0.56	0.784
**heart**	0.33	0.017	0.8	0.264
**lung**	0.536	0.017	10.482	5.6184
**skin**	2.6	0.017	0.1	0.26
**tendon**	3	0.017	0.01	0.03
**other**	5.524	0.017	0.02	0.1104
**bone**	4	0	0	0
**adipose**	17.5	0.8	0.0422	0.7385

**Total**	70			5.5877

The only PBPK features that must be uniquely specified for propofol are the tissue/blood partition coefficients. The free (unbound) water concentration (c, amount/liter water) plays a central role in the PBPK calculations (see [6] for details). For example, at equilibrium, c will have the same value in all tissues and blood and the total (measured) concentration (C, amount/kg) is related to c by:



where v_w_^i ^(liters/kg) is the water fraction in tissue i and fw_i _is the fraction of the total solute that is free in the water phase. The parameter fw_i _characterizes the equilibrium tissue/blood partition coefficients:



The tissue/blood ratio determines the "effective" volume of the different tissues and the overall volume of distribution.

As discussed in the Background section, it is assumed that the propofol tissue concentration depends simply on the propofol partition in the tissue water and fat:



where v_w_^i ^and v_f_^i ^are water and fat fractions in tissue i, c^i ^is the free water concentration, C_f _is the fat concentration and K_oil _is the oil/water partition coefficient (= C_f_/c). The values used for the tissue fat fraction (v_f_^i^, Table 1) are identical to those used in the previous application of PKQuest to the pharmacokinetics of the volatile anesthetics [4]. They were determined from in vitro measurements of human tissue/air partition coefficients for a number of volatile anesthetics with a wide range of oil/air and water/air partition. [See Additional file 1 for a description of the experimental basis for this set of values of v_f_]. The value used for K_oil _of propofol was 4715, which was determined by Weaver et. al. [1] from measurements of the water/Diprivan partition and the triglyceride concentration in Diprivan. This is similar to the value of the octanol/water partition of 4300 determined by Tonner et. al. [27] and smaller than the octanol/water partition of 6165 reported by Hansch et. al. [28]. Triglyceride should provide the best model for tissue lipid/water partition.

The value that is used in eq. (4) for the propofol fraction that is free in the blood (fw_blood_) is based on experimental measurements of the fraction free in human plasma (freepl), which is related to fw_blood _by:



where rblpl is the blood/plasma concentration ratio. In normal subjects, freepl varies from subject to subject by about ± 40% of the mean [14-19], primarily because of individual variation in plasma lipids. It is assumed that only the plasma lipid varies from subject to subject, while the red cell lipid is constant, so that the value of the blood/plasma ratio (rblpl) also varies for each subject as a function of freepl:



where rblpl_st _and freepl_st _are the mean experimental human propofol values for the blood/plasma ratio and the free plasma concentration and hmt is the standard hematocrit.

To summarize the procedure that is used to determine the tissue blood/partition coefficients for the PBPK model as a function of the PBPK parameter freepl: 1) The blood/plasma ratio (rblpl) is determined from eq. (7) using the experimental values for freepl_st _and rblpl_st _(see below). 2) fw_i _for each tissue is determined using eq. (5) and the standard values of K_oil _and v_f_^i^. 3) fw_blood _is determined from eq. (6). 4) Finally, the tissue/blood partition is determined from these values of fw_i _and fw_blood _using eq. (4). Since the plasma propofol concentration was measured in the experiments of Schnider et. al [20], the whole blood model concentrations were first converted to the equivalent plasma concentration before the model results were output in the plots of plasma model concentration versus experimental data.

### Standard value for human propofol free plasma concentration fraction (freepl_st_) and blood/plasma ratio (rblpl_st_)

As discussed above, the tissue/blood partition depends on the value of the fraction free in plasma (freepl). Experimental measurements of the normal human free plasma propofol fraction (freepl) fall into two different ranges. The smaller value of about 0.01 comes from a series of publications by Suarez and colleagues using ultrafiltration [14-16]. A larger value of about 0.02 has been reported by several other research groups: Using equilibrium dialysis, Altmayer et. al [17] reported values ranging from 0.014 to 0.026 and Servin et. al. [18] reported an average value of 0.022; while Mazoit and Samii [19] report an average value of about 0.02 using a charcoal co-binding technique. All reports agree that the plasma binding is independent of propofol concentration in the clinical concentration range and that the normal individual variation is about ± 40% of the mean. Although the difference in these measurements of the freepl_st _(0.01 versus 0.02) is small in absolute value, it has a dramatic effect on the pharmacokinetics – producing a roughly two-fold difference in the tissue/blood partition coefficient (eq. (4)). Since the PBPK analysis (see below) is consistent with a value of about 0.022, this was the value that was assumed for standard values for freepl (freepl_st_). The reported values for the normal human blood/plasma ratio (rblpl_st_) vary from 1.1 to 1.3: [18,19,29,30] and a standard value of the blood/plasma ratio (rblpl_st_) of 1.0 was assumed.

Because of the large (40%) individual variation in freepl, it might be regarded as an adjustable parameter that varied from subject to subject. However, since it was found that there was no significant improvement of the PBPK model fit to the individual data when freepl was allowed to vary, in the following analysis it is assumed that all subjects had a freepl of 0.022. Using eqs. (4) – (7), the corresponding normal model values for the tissue/blood partition coefficients using the value of v_f_^i ^in Table 1 are: adipose 84; brain 1.87; liver 2.12; intestine 1.7 and the rest 1.45. These values are in the same range as the experimental measurements of Weaver et. al. [1] after a 2 hour constant infusion in sheep: brain 1.8 – 2.4; kidney 1.36 – 1.85; skeletal muscle 0.68 – 1.4 (the range corresponds to the values for 2 different infusion rates). Assuming that the propofol binding in blood is produced entirely by partitioning between the blood lipid and water, the freepl value of 0.022 corresponds to a value for the fraction of lipid in whole blood (v_f_^blood^) of about 0.0093 (using eq. (5)), which is in the range of the normal human blood total lipid (total plasma lipid of 0.0082 gm/ml [31] and blood/plasma lipid ratio of 1).

### PBPK description of propofol metabolism

It is assumed that the kinetics of all the tissues are described by a flow limited, well mixed model, except for the liver, which is described using the "Dispersion" model of Rowland and colleagues [32,33] with a dispersion coefficient of 0.3. Measurements of brain, arterial and venous propofol concentrations during infusions in sheep are consistent with a flow limited, well mixed model for the brain [34]. This well-mixed tissue assumption is only correct as a first approximation and there is some evidence that it may not be rigorously correct for muscle [35].

Since the kinetic analysis of Schnider et. al. [20] of the data analyzed in this paper indicated that the kinetics were linear over the constant infusion range (25 to 200 μg/kg/min) that was investigated, a linear PBPK model is used. It is assumed that the propofol removal is entirely the result of liver metabolism and is described by the intrinsic liver clearance (Tclr), defined by:

(8)     *Q(x)dx = Tclr c(x) dx*

where Q is the rate of liver metabolism (amount/min) and c is the free liver tissue concentration. Since a dispersion model is used for the liver [32,33], the metabolism varies as a function of distance (x) from the start of the liver sinusoid. For the subjects investigated in this paper, Tclr varies from about 65 to 400 liters/min/70 Kg human. Although Tclr has units of clearance it is not equal to the actual liver whole blood clearance for two reasons. First, the concentration term in eq. (8) is the free liver concentration, not the whole blood concentration. The effective clearance from the blood (Tclr_blood_) is related to Tclr approximately by:

(9)     *Tclr_blood _= (water / blood partition) Tclr *≈ 0.02 *Tclr*

**Figure 5:**
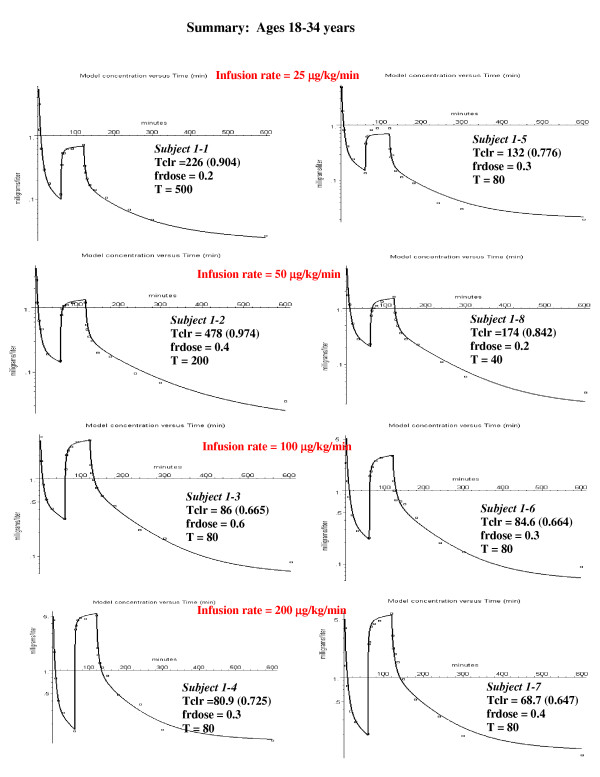
Individual PBPK model fits to the experimental data for the young subjects. The values of the 3 parameters that provided the best fit for each individual subject are listed: Tclr = intrinsic liver clearance; frdose = fraction of the bolus dose sequestered in first passage through the lung; T = time constant for release of sequestered propofol. The value in parenthesis following Tclr is the fraction of the total liver blood flow that is cleared (eq. (10)).

**Figure 6:**
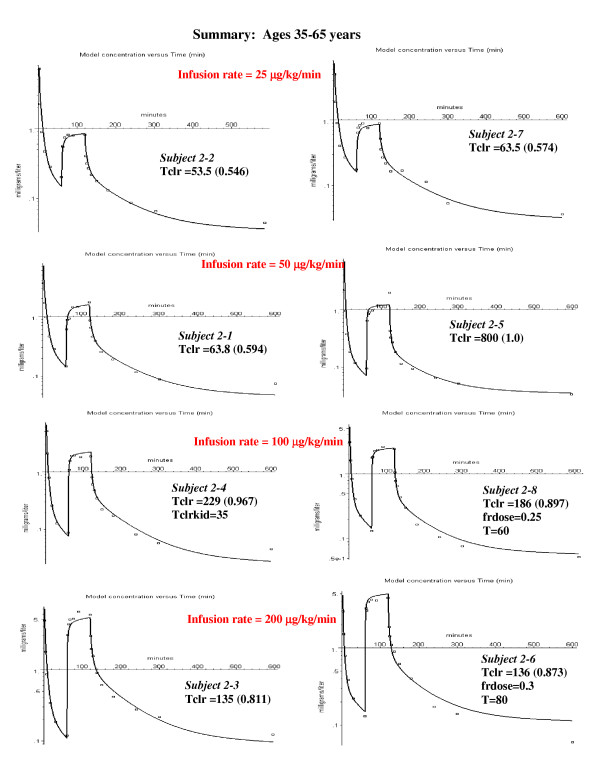
Individual PBPK model fits to the experimental data for the middle aged subjects. The values of the 3 parameters that provided the best fit for each individual subject are listed (see fig. 5). For 6 of the 8 subjects there was no pulmonary sequestration of the bolus dose. In order to fit the data for subject 2–4, a small amount of extra-hepatic metabolism was required (kidney clearance = Tclrkid = 35).

**Figure 7:**
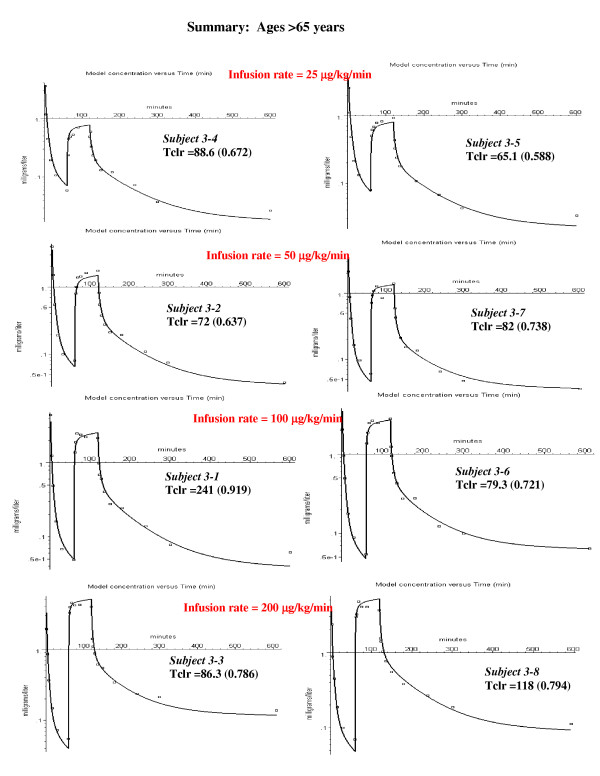
Individual PBPK model fits to the experimental data for the old subjects. No pulmonary sequestration of the bolus dose was required in any subject in this age group. Therefore, there is only 1 adjustable parameter (intrinsic liver clearance, Tclr).

A second, more complicated, correction arises from the position dependence in eq. (8) and the fact that as Tclr increases to infinity; the actual liver clearance rises to a maximum equal to the liver blood flow. A more direct measure of liver metabolism is the steady state "Fractional Liver Clearance" defined by:



Table 2 provides a useful conversion between Tclr and Fraction Liver Clearance for the standard human. In the description of the PBPK model fits to the individual subjects (figs. 5, 6, 7), both Tclr and Fraction Liver Clearance are indicated. In PKQuest, either Tclr_liver _or the equivalent Fraction Liver Clearance can be input.

**Table 2:** Relationship between the intrinsic liver metabolic clearance (Tclr) and the fractional clearance of the total liver blood flow or the absolute steady state liver blood clearance for a standard male (20% fat, fraction free in plasma = 0.02).

**Tclr (liters/min)**	**Fraction Liver Blood Flow**	**Liver Clearance (liters/min)**
10	0.13	0.23
50	0.47	0.846
100	0.68	1.224
200	0.86	1.548
300	0.93	1.674
400	0.962	1.731
500	0.977	1.76

Although there is evidence of extrahepatic human propofol metabolism [36], it has been assumed in the PBPK model that all the clearance results from liver metabolism. With the exception of subject 2–4, the clearance could be modeled by this assumption for all the subjects in the study- that is, the clearance did not exceed the total liver blood flow. For subject 2–4 (see fig. 6) an additional extrahepatic (about 10%, assigned arbitrarily to the kidney) metabolism was required to fit the data.

It has been assumed in the PBPK model that there is no pulmonary propofol metabolism. This is supported by the experiments of He et. al. [37] who found no significant pulmonary artery – radial artery propofol concentration difference during a constant infusion. Also, Gray et. al.[36] found no arterial – venous difference in propofol or propofol metabolites during the anhepatic phase of liver transplantation. However, in opposition to these experiments, Dawidowicz et. al. [38] found a significant pulmonary arterial – venous difference for both propofol and propofol metabolites, indicating some pulmonary metabolism.

### Pulmonary sequestration

As discussed in the Background section, the PBPK analysis suggests that in some subjects a fraction of the bolus propofol injection is sequestered in the lung and then slowly released. This sequestration is described by two parameters: 1) frdose – the fraction of the dose that is sequestered; and 2) T – the time constant of the exponential release from the lung of the sequestered fraction. The rate of release from the lung (R(t)) of the sequestered propofol after the bolus input is described by:

(11)     *R_bolus _*(*t*) = (1/*T*) *Dose frdose *exp(-*t / T*)

This sequestration was incorporated into the PBPK model simply by dividing the bolus input into two non-sequestered components: 1) A bolus input of Dose × (1-frdose); and 2) An exponential input of total amount = Dose × frdose.

The data was also analyzed to check whether there was sequestration of the constant infusion dose. This required a more complicated modification. The constant infusion was again divided into two components. The first, non-sequestered, component was just a constant infusion of Dose × (1-frdose). For the second, sequestered component, it was assumed that a fraction frdose of the constant infusion was accumulated in a well-mixed compartment in the lung that was released with the same exponential time course as the bolus dose. The release for the bolus dose (eq. (11)) corresponds to the bolus response function of this sequestering lung compartment. Thus, the rate of release (R_I_(t)) for an arbitrary input I(t) to this sequestering compartment is equal to the convolution of I(t) and R_bolus_(t):



This input is pre-programmed into PKQuest as one of the input options and is invoked simply by calling this input option.

### Determination of PBPK parameters

Each subject in the study was given a bolus propofol injection followed 60 minutes later by a constant 60 minute infusion and the PBPK model was used to fit the individual concentration curves. The previously determined "standard human" PBPK parameter set (organ blood flow, volume, etc., see Table 1) was used for all subjects. The tissue/blood partition was determined from eqs. (4)–(7) using a freepl = 0.022, a blood/plasma ratio of 1 and the tissue lipid fractions determined previously (Table 1) [4]. The values of these standard parameters depend on the percent body fat, which is determined using eq. (2).

For each subject the intrinsic liver clearance was adjusted to fit the data. For some subjects, in order to fit the bolus phase it was also necessary to choose the two parameters describing the pulmonary sequestration (frdose and T). Several steps were used to determine these parameters for each subject: 1) Subjectively adjust the liver clearance (Tclr) to fit the constant infusion phase. 2) If both the bolus and constant infusion phase could be adequately fit by one value of the liver clearance, than it was assumed that there was no pulmonary sequestration and step 3 was implemented. In 14 of the 24 subjects, no pulmonary sequestration was required. In the other 10 subjects it was necessary to adjust frdose and T to fit the bolus phase. Depending on the value of T, the slow release from the sequestered compartment can extend into the constant infusion phase, and it was necessary to repeat this cycle of parameter estimation. 3) Finally, a 1-parameter Powel non-linear minimization routine in PKQuest was used to find the value of Tclr that provided the best fit to the entire experimental concentration curve (bolus plus constant infusion) for that subject. This used a weighted least square minimization technique with weights determined by assuming that the standard deviation of the measurement was proportional to the model concentration.

The experimental plasma concentration was collected over a time course of 0 to 600 minutes, with the first data points at 1, 2, 4 ... minutes and the last two samples at 300 and 600 minutes. Because of mixing and circulation time effects [39], the PBPK model is not accurate at times < 2 minutes, so that the first point that was used in the analysis was at 2 minutes. The fits to the 600 minute data was less accurate than for the earlier data points (see figs. 5, 6, 7). The 600 minute data point has the most scatter because the status of the subjects was not controlled during the 300 to 600 minute time period and ambulation or food intake during this period could produce large shifts in the PBPK parameters, particularly muscle, liver and fat blood flow. The quality of the fits were quantitated and compared with the 6 parameter NONMEM fit by the use of the "weighted residual error" (WRE) defined as the average value of the absolute (measured – model)/model concentration ratio

All the calculations and the graphical output were implemented using the PKQuest software routine. The procedures involved in using PKQuest have been described previously [3-8]. All of the standard human PBPK parameters (blood flows, organ weights, etc) and the equations for, e.g., converting freepl to tissue partition values, are pre-programmed and do not have to be entered. The only parameters that the user must enter are those that are unique to the propofol study: the bolus input and constant infusion rate; the experimental arterial plasma concentration data; the PBPK parameters weight, height, sex, age, and Tclr, and, for subjects with pulmonary sequestration, frdose and T. All of the figures used in this paper represent standard PKQuest graphical output. [See Additional file 1 for a sample PKQuest Maple worksheet for one subject].

### Model simulation of normal weight and obese subjects

The PBPK model parameters were chosen to simulate the experiments of Servin et. al. [40] in which the pharmacokinetics of normal weight and morbidly obese subjects were compared. The average values were used for the fraction free in plasma (freepl = 0.022) and intrinsic liver clearance (Tclr = 162 liters/min/70 kg). This value of Tclr corresponds to a steady state liver clearance of 1.49 liters/min or 83% of the total liver flow for the standard 70 kg man (see Table 2). Results were compared for normal weight subjects (fat fraction = 20%) and for the average obese subject studied by Servin et. al. Using the reported body weight and ideal body weight of the obese subjects, it was estimated that they had a fat fraction of about 50% based on the regression relationship of Rhode et. al [41] that 69% of the weight greater than ideal weight is fat.

One significant change was made in the PBPK parameters for the obese subjects. Adipose blood flow is heterogeneous, with the highest values in subcutaneous tissue and lower values in visceral and perirenal fat [42]. It is probable that morbidly obese subjects have a lower average adipose blood flow then normal weight subjects. An estimate of the adipose blood flow can be obtained from the relationship between cardiac output and excess body weight. The cardiac output measurements in morbidly obese subjects of Alexander et. al. [43] were used to estimate an average adipose tissue blood flow of 0.03 liters/kg, 28% less than the standard value of 0.042 liters/kg (see Table 1). All the other PBPK parameters are identical for the normal and obese subjects. Since it is assumed in PKQuest that liver weight is a constant fraction of non-fat body weight, the obese subjects have a lower relative liver weight and, therefore, a lower rate of propofol clearance per kg body weight. Servin et. al. [40] used a stepwise infusion regimen: 0.35 mg/kg/min for 5 minutes, 0.2 mg/kg/min for 10 minutes, and 0.1 mg/kg/min for the remainder of the 180 minute infusion. In the simulation, a constant 0.1 mg/kg/min infusion for the entire 180 minutes was assumed. The same propofol infusion rate/kg was used in both normal and obese subjects.

PKQuest is freely distributed at 

## Results

### Pulmonary sequestration

**Figure 2:**
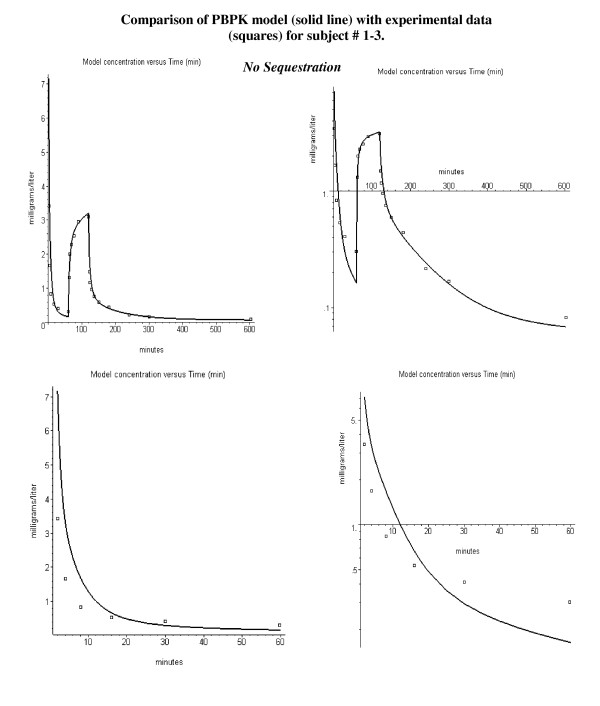
Error in fitting the kinetics following the bolus injection (0 to 60 minutes) using the PBPK parameters determined by fitting the constant infusion kinetics (>60 minutes) assuming no pulmonary sequestration. The solid lines are the PBPK model predictions and the squares are the experimental results of Schnider et. al. [20]. The top row shows the entire time course and the bottom shows the first 60 minutes. Absolute concentration on left and semi-log plot of concentration versus time on right.

Figure 2 shows a comparison of the PBPK model versus the experimental data for the arterial plasma concentration for subject 1–3. The figures on the left are plotted on an absolute scale, and those on the right on a semi-log scale. The bottom row shows the data for just the bolus phase (0 to 60 minutes). All subjects in the first two age groups received the same initial bolus propofol dose (2 mg/kg), followed 60 minutes later by a 60 minute constant infusion at rates varying from 25 to 200 μg/kg/min. Subject 1–3 had a constant infusion rate of 100 μg/kg/min, which is large enough to swamp out most of the residual from the initial bolus. In fig. 2 the liver clearance (Tclr) has been adjusted to optimize the fit to the constant infusion phase (60 to 600 minutes).

It can be seen in fig. 2 that, using a value of Tclr that accurately described the constant infusion phase, the model prediction for the bolus phase is poor. The deviation between the model and bolus data is unusual – with too high a model concentration at early times (2, 4, 8, and 16 minutes) and too low a concentration at the 30 and 60 minutes time points. This same discrepancy in the model prediction was noted in the original analysis of this data by Schnider et. al.[20] using a NONMEM compartmental model. It is impossible to explain this deviation using the standard PBPK model. Any variation that improves the fit to the bolus phase significantly worsens the fit to the constant infusion phase.

**Figure 3:**
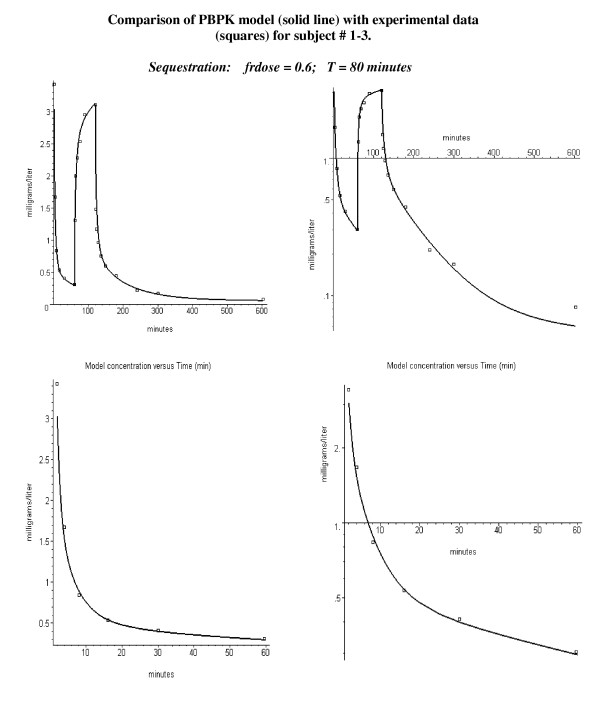
Same experimental data as in figure 2 except that 60% of the bolus dose has been sequestered in the lung and released with a time constant of 80 minutes.

This deviation between theory and experiment could be explained if part of the bolus dose was sequestered in the lung and then slowly released. The initial sequestering would reduce the blood concentration at the early times, while the later release would increase the concentration at later times. There is direct experimental support for human pulmonary sequestration of the lipid emulsion that is used in the propofol formulation [37,44-46] see Discussion). Figure 3 shows that the experimental data for the entire time course for subject 1–3 can be accurately fit by the PBPK model if it is assumed that 60% of the bolus dose is sequestered (= frdose) and then released with an eighty minute time constant (T, see, eq. (11)).

**Figure 4:**
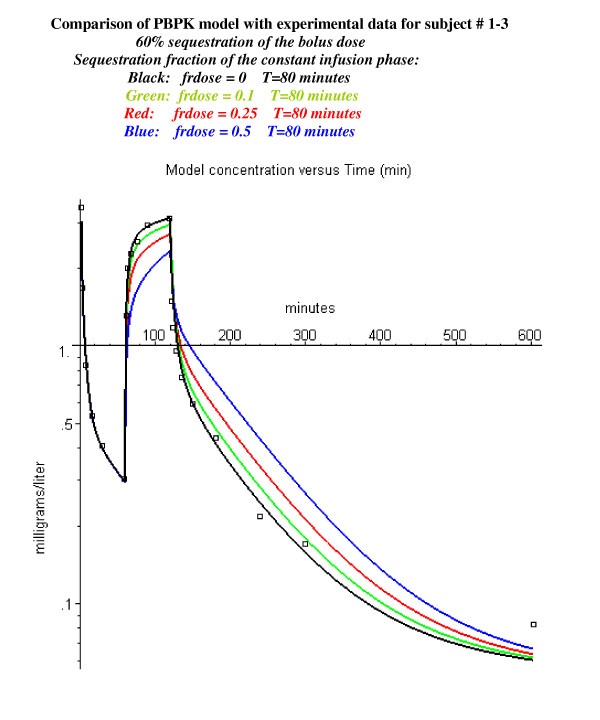
Effect of pulmonary sequestration of 0, 10, 25 or 50% of the continuously infused dose. Same experimental data as in figure 2 with 60% sequestration of the bolus dose. The sequestered propofol is released with a time constant of 80 minutes.

The PBPK model was modified to look at whether the constant infusion phase dose was also sequestered with the same time constant (see Methods, eq. 9). Figure 4 shows the fit for this same subject with a sequestered fraction of 0.5 (black line), 0.25 (red line) or 0.1 (green line) during the constant infusion phase. (The sequestered fraction during the bolus phase is 0.6). It can be seen that sequestering as little of 10% of the constant infusion dose significantly worsens the prediction of the PBPK model – suggesting that there is no sequestration during the constant infusion phase.

This subject (#1–3) was chosen as an illustration because he had the largest value of sequestration. However, all the other subjects in the younger age groups had some sequestration (20 to 40%, fig. 5), as indicated by an improvement in the fit to the bolus phase. In the middle aged group, only two of the subjects had significant sequestration (fig. 6). The subjects in the oldest group received a bolus dose one half that of the two younger age groups, and none of the subjects in the oldest age group had significant sequestration (fig. 7).

### PBPK analysis for 18–34 year old subjects

Figure 5 shows the semi-log plots of the PBPK arterial plasma concentrations for the 8 young subjects at the 4 different constant infusion rates (all subjects received the same bolus dose). It can be seen that the PBPK model provides a good description of the experimental results for the 8 subjects over the entire range of infusion rates. The values of the 3 adjustable PBPK parameters are listed in the figure. The value of the intrinsic liver clearance (Tclr, eq. (8)) ranges from 85 to 478 liters/min. As discussed above (see Table 2), the actual liver clearance has a very non-linear dependence on Tclr. The value in parenthesis after Tclr in fig. 5 is the steady state fractional liver clearance. This clearance provides a better indication of the individual variation in liver clearance. The value of the fraction of the bolus dose that is sequestered (frdose) varies from 0.1 to 0.6, with 5 of the 8 subjects having a frdose 0.2 – 0.3. The value of the time constant for release of the sequestered propofol (T) varies from 40 to 500 minutes with 5 of the 8 subjects having a value of 80 minutes. The fits are not very sensitive to the value of T, and differences of ± 30 minutes are not significant. The average weighted residual error for these subjects is 13.5% (Table 3).

**Table 3:** Comparison of the "average weighted residual" percent error for the PBPK model (this paper) and the 6 parameter NONMEM model of Schnider et. al. [20]. The PBPK model fits were for both the bolus and the constant infusion input phases, while the NONMEM model fit was for just the constant infusion phase. The PBPK model results are subdivided into the different age groups. The column labeled "Individual Fits" is the error when the model parameters were adjusted for each subject (see figs. 4–6). The column labeled "Average Fits" is the error when one parameter set with a linear age dependence for the fraction of pulmonary sequestration (eq. (13)) was used for all subjects (see figs. 9–11). The "Average Fit" error for the NONMEM model is listed for the case where the same set of 6 parameters were used for all subjects ("No Covariates") or an additional 5 covariate parameters (e.g. weight, height, age, etc) were used ("Covariates").

**PBPK Model Bolus + Infusion**	**Age Group**	**Individual Fits**	**Average Fits**
	18–34 years	13.5%	18.5%
	35–65 years	16.1%	23.8%
	>65	15.4%	17.6%
	*Average*	*15.0%*	*20.0%*
			
NONMEM Model Infusion Only [20]		*14.18%*	*23% *No Covariates *17.39% *5 Covariates

### PBPK analysis for 35–65 year old subjects

Figure 6 shows the semi-log plots of the PBPK model for the 8 middle-aged subjects at the 4 different constant infusion rates. Pulmonary sequestration of the bolus dose was decreased for this age group. In 6 of the 8 subjects, the fits were only slightly improved by adding sequestration, and, to minimize the number of adjustable parameters, it was assumed that sequestration was negligible. A sequestered component is clearly present only for the two subjects 2–6 and 2–8. The propofol kinetics for the other 6 subjects in fig. 6 can be satisfactorily fit by a PBPK model with just one adjustable parameter – Tclr. The range of values for both these parameters are similar to those in the younger age group (fig. 5). For one subject in this group (# 2–4) it was necessary to add an additional renal clearance in order to fit the data. The weighted residual error is 16.1% (Table 3).

### PBPK analysis for subjects older then 65 years

Figure 7 shows the semi-log plots of the PBPK model for the 8 subjects in the >65 year old group at the 4 different constant infusion rates. These subjects were given a bolus dose of 1 mg/kg, half the value of the bolus dose for the younger subjects. Since there was no evidence of pulmonary sequestration in any of these 8 older subjects, the PBPK model required only one adjustable parameter (Tclr). The weighted residual error was 15.4% (Table 3).

### Age dependence of PBPK parameters and model predictions using "averaged" parameters

**Figure 8:**
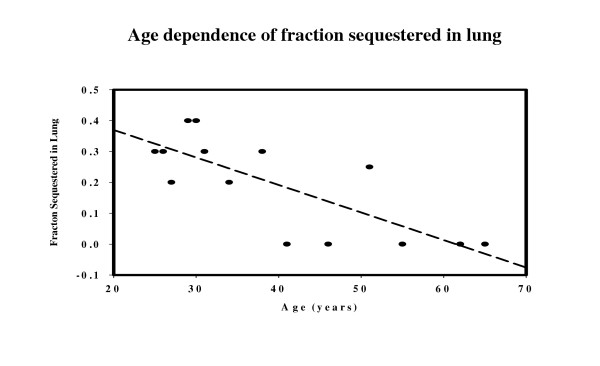
Age dependence of the fraction sequestered in lung. The dashed line is the linear regression for the data (eq. (13)).

Table 4 lists, for the 3 age groups, the average values of the age, steady state fractional liver clearance, the intrinsic liver clearance (Tclr) and the fraction of the bolus dose sequestered in the first pass through the lung (frdose). There is no significant dependence of liver clearance on age. The fraction sequestered (frdose) decreases with age and a linear dependence was determined (see fig. 8).

**Table 4:** Age dependence of steady state fractional liver blood flow clearance (Liver Clearance), the intrinsic liver clearance (Tclr), and fraction of bolus dose sequestered in lung (frdose).

**Age (years)**	**Liver Clearance**	**Tclr (liters/min)**	**frdose**
29.125	0.772	165	0.3
52.5	0.781	208	0.06875
74.75	0.762	104	0



The oldest subject group (>65) was not used in this frdose correlation calculation because they received only half the bolus dose of the other subjects and there was no significant sequestration (see Discussion).

**Figure 9:**
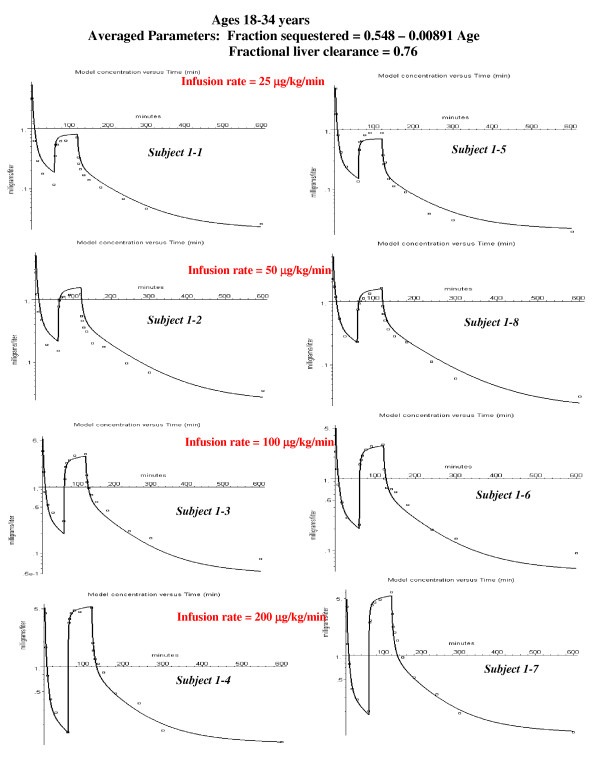
PBPK model fits to the experimental data for the young subjects using the same age dependent parameters for all subjects: fractional liver clearance = 0.76; fraction sequestered described by eq. (13), and T = 80 minutes.

**Figure 10:**
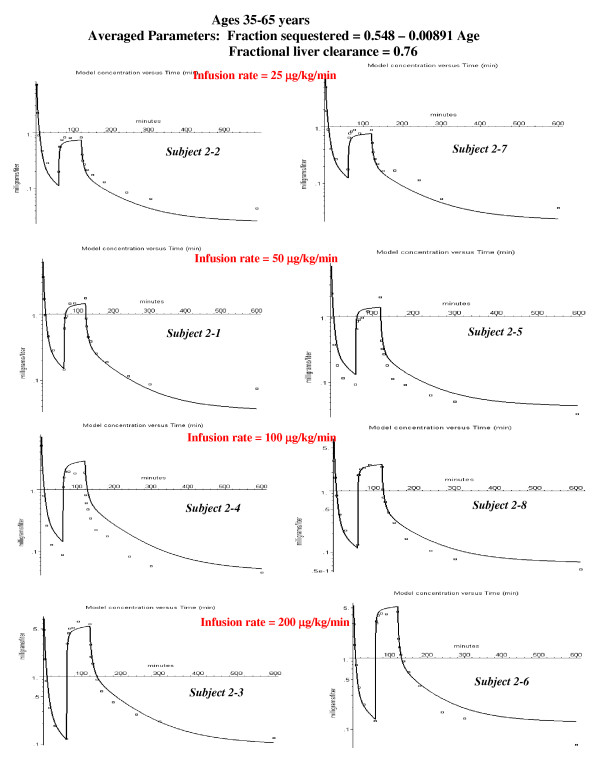
PBPK model fits to the experimental data for the middle-aged subjects using the same age dependent parameters for all subjects (see fig. 9).

**Figure 11:**
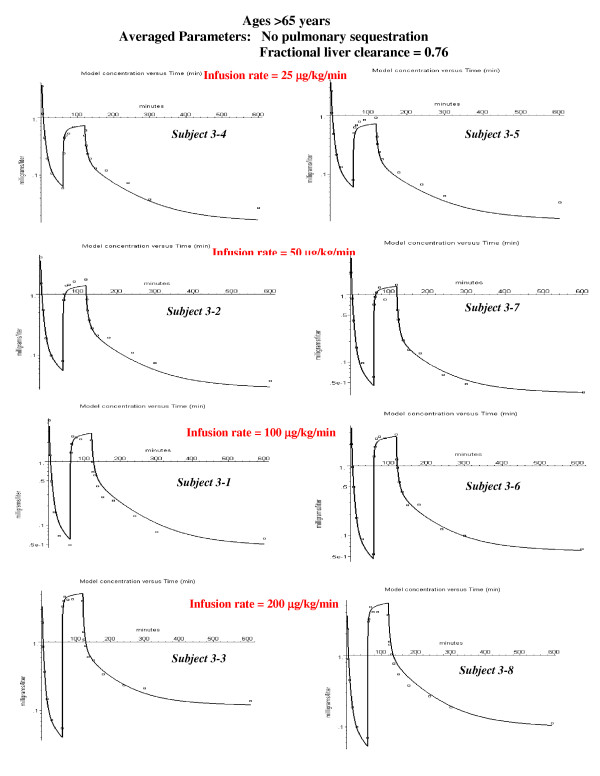
PBPK model fits to the experimental data for the old subjects using the same age dependent parameters for all subjects (see fig. 9).

Figures 9, 10, 11 show the accuracy of the PBPK model predictions when just one set of "averaged" PBPK parameters is applied to all the subjects. The "averaged" parameters are: 1) Fractional liver clearance = 0.76; 2) Sequestration time constant = 80 minutes; and 3) fraction pulmonary sequestration described by eq. (13). The weighted residual error using these "averaged" values is listed in Table 3.

## Discussion and conclusions

### Pulmonary sequestration

The pharmacokinetic evidence for this sequestration comes from a comparison of the kinetics after the bolus injection versus a constant infusion. It was clearly recognized in the original analysis of this data by Schnider et. al. [20] that there was some systematic difference between the kinetics for these two different inputs. Schnider et. al. fit the constant infusion phase kinetics for each subject with a 6 parameter NONMEM model. When these kinetic parameters for the infusion phase were used to fit the initial bolus kinetics, the predicted concentrations for the early time points (2 to 4 minutes) were about 50% greater then the experimental values, while the predicted concentration for the later data points (>8 minutes) were about 50% less than the experimental values. The same systematic difference is seen in the younger subjects when the PBPK parameters for the constant infusion phase are used to predict the concentration following the bolus dose (fig. 2).

Pulmonary sequestration of the bolus dose with a slow release provides an explanation of this systematic difference. The sequestration will reduce the concentration of the early time points and, as the sequestered dose is slowly released, increase the concentration of the later time points. A simple model in which a fraction (frdose) of the bolus dose is sequestered and is then released as a single exponential (eq. (11)) provides a good fit to the experimental data (fig. 3, and figs 5, 6, 7). The fraction of the dose that was sequestered was largest in the younger age group, ranging from 0.2 to 0.6 (fig 5).

As described in fig. 4, there does not seem to be any significant sequestration when the propofol is given as a constant infusion because its subsequent slow release would produce a significant deviation of the model predictions from the observed experimental data. This suggests that the sequestration depends on the concentration of the emulsion when it is mixed with the venous blood at the injection site. The bolus injection rate of 140 mg/70 kg/20 sec propofol in Diprivan corresponds to an injection of about 4.2 gm/min of lipid into the vein of a 70 kg man. In contrast, the highest rate of constant infusion (14 mg/70 kg/min) represents a 30-fold lower infusion rate.

There is direct experimental evidence for pulmonary sequestration of propofol following a bolus injection in humans [37,45]. He et. al.[37] simultaneously injected propofol and indocyanine green (ICG) in a central vein and sampled radial arterial blood at 1 second intervals for a period of 1 minute – the first pass time. They injected 5 mg/70 kg propofol within 1 second – about twice the rate of the bolus injection of Schnider et. al. [20] that was fit with the PBPK model. A first pass pulmonary clearance of propofol of about 28% was estimated from the difference in the first pass AUC of propofol and ICG. This analysis cannot distinguish between pulmonary metabolism versus sequestration. However, in the same study, He et. al. [37] demonstrated that there was no significant difference in the radial and pulmonary artery concentrations during a 60 minute constant infusion, indicating that propofol is neither metabolized nor sequestered during a constant infusion.

In the standard propofol formulation (Diprivan), the propofol is dissolved in a lipid emulsion that is identical to the emulsion (Intralipid) that is used for parenteral feeding. There is also direct evidence that Intralipid is sequestered in the human pulmonary circulation. Zauner et. al. [44] measured the brachial artery – pulmonary artery concentration difference of Intralipid during and after a constant infusion. At high rates of Intralipid infusion (about 6.5 times the maximum rate used by Schnider et. al. [20]) they observed a significant pulmonary sequestration of about 20% during the infusion, with a subsequent release of the sequestered Intralipid that persisted for at least 15 minutes after stopping the infusion. In addition, Gigon et. al. [46] found fat in the lumen of pulmonary capillaries in human lung biopsies a few minutes after beginning Intralipid infusion.

There are large individual variations in the model predictions of the fraction of the dose that is sequestered, varying from 0 to 60%. All of the younger subjects had some sequestration (fig. 5) with a median value of about 30%. Only two of the eight middle-aged subjects (fig. 6) and none of the subjects in the oldest group (fig. 7) had significant sequestration. This variation cannot be explained by experimental variations in the bolus infusion rate, which was carefully controlled. The lack of sequestration in the oldest subjects might be explained, in part, because they received only half the bolus dose of the other two groups. Variation in sequestration could result in significant variations in the pharmacodynamic effect of a bolus propofol injection.

This new evidence supporting the concept of pulmonary sequestration is indirect since it is based just on an analysis of the PBPK model. There clearly is a need for more direct experimental measurements to either confirm or rule out this effect. In addition, the predicted differences in the magnitude of the sequestration between the young and middle-aged subjects is a surprising result and one that requires further documentation.

### Validity of the PBPK propofol model

The PBPK model (fig. 1) requires assumptions about a large number of physiological parameters, such as tissue volumes and flows, and is limited by many simplifying assumptions, such as that the tissue regions are well stirred (except for the liver) and flow limited. The usual criticism of the PBPK approach is that, since it uses a large number of adjustable "physiological" parameters that cannot be directly measured, it becomes, in effect, a glorified compartmental model. This criticism applies particularly to the tissue/blood partition coefficients of the different organs, which are impossible to directly measure in humans. The major goal in the development of PKQuest is to address this criticism by the development of a "standard human" PBPK data set whose values have been refined by application to more than 25 different solutes with a wide range of pharmacokinetic properties [3-6,8].

This propofol PBPK analysis uses the previously determined "standard human" data set for the organ volumes and blood flows so that these parameters are no longer "adjustable". Because of the very high propofol fat partition, the tissue/blood partition is dominated by the partition into the tissue and blood fat. In this PBPK analysis, the tissue/blood partition was directly determined using the tissue fat fractions that were derived in a previous PBPK analysis of the volatile anesthetics [4] along with the experimental value for the propofol fraction unbound in blood (freepl = 0.022), and no other assumptions or adjustable parameters were required. Thus, in this PBPK model there is only one new adjustable parameter required to completely describe the blood concentration curves – the intrinsic liver clearance. (Two additional parameters are required to describe the sequestration of the bolus dose in some subjects).

The very high fat solubility of propofol makes it an ideal candidate for a PBPK model. The partition of propofol in tissue fat dominates the tissue/blood partition coefficient, allowing one to estimate the tissue/blood partition simply from knowledge of the tissue fat fraction. For other less fat soluble solutes, the tissue/blood partition cannot be predicted by this a priori approach, and the number of poorly characterized adjustable PBPK parameters is markedly increased.

### Comparison of PBPK and compartment models

Although compartmental and PBPK models are often regarded as competitors, they are actually complementary and serve different purposes. Compartment models, as implemented in NONMEM, provide an unbiased parametric description of a data set using a using a minimal number of model assumptions. If one is only interested in a parametric description of a given clinical data set, then this is all that is needed. The limitation of the compartment model is that the parameters are only weakly related to physiological variables. One cannot use a compartment model to predict the pharmacokinetics under varying physiological conditions, such as changes in portal, muscle or fat blood flow, or variations in plasma protein binding or body fat content.

This paper describes one of the few quantitative comparisons of PBPK and compartmental models that is available in the literature. Table 3 compares the average weighted residual percent error (WRE) for each subject using the 6 parameter NONMEM compartment model versus the 1 parameter PBPK model (for some subjects 2 additional parameters are required to fit the bolus phase). The WRE for the compartment model are for the fits to just the constant infusion phase, while the WRE for the PBPK model is for both the bolus and infusion phase. The quality of the fits for the PBPK model is slightly poorer than that for the NONMEM compartmental model (compartment model: 14.18% error; PBPK model: 15.0% error).

The clinically important measure of the quality of the fit is how well it predicts the kinetics using "averaged" parameters without any prior information about the individual model parameters. Two different "averaged" errors were reported for the NONMEM model (Table 3). The error was 23% when an identical "optimal" set of 6 parameters was used for all subjects. The error was reduced to 17.39% when 5 "covariate" parameters were used that were based on the subjects' sex, weight, height and age. The ability to calculate these covariate parameters is one of the major strengths of NONMEM compartmental modeling. Although some PBPK models have been developed that allow Bayesian population modeling [47], this is not possible with the current version of PKQUEST. Thus, it is not possible to determine the covariate dependence of the PBPK model parameter (Tclr, frdose, T) on subject's age, weight, etc. The only correlation that was investigated for the PBPK model was the age dependence of the parameters (Table 4, fig. 8). The only parameter with significant age dependence was the pulmonary sequestration fraction (fig. 8). Using this linear age dependent correlation (eq. (13)) an "averaged" PBPK model fit was obtained for all subjects (figs. 9, 10, 11). These calculations also include a limited correlation for sex, age, weight and height through the use of an estimate of each subject's percent body fat using eq. (2). For the PBPK model, the "average" error was 20.0%, significantly worse than the covariate NONMEM model error of 17.39%. It should be emphasized that the PBPK model error is for both the bolus and constant infusion phases, while the NONMEM error is for just the constant infusion phase.

An important limitation of the NONMEM compartmental approach is that, since it is only a parametric description of a particular data set, errors may occur when the model is extrapolated beyond this data set. This is clearly illustrated for propofol if one tries to extrapolate the compartmental model to times longer than those used in the experimental data set (10 hours). The time constant (T) for equilibration with adipose tissue is described by:



For an adipose blood flow of 0.042 ml/gm/min and an adipose/blood partition coefficient of 84, T is 2000 minutes, or 33.3 hours. Accurate estimates of steady state clearance (Cl_ss_) and volume of distribution (V_ss_) require that the kinetics be determined for times of about twice that of the longest time constant, eg. about 50 hours. Most kinetic studies are for periods much shorter than this and have led to misleading values for these parameters. This is clearly illustrated by the NONMEM compartmental analysis of Schnider et. al. [20]. Based on measurements for a 10 hour sample period, they found a V_ss _of about 260 liters, about 10 times less than the V_ss _of 1200 to 3940 liters obtained by Campbell et. al. [48], Morgan et. al. [49] and Albanese et. al. [50] using plasma values sampled for times varying from 40 to 100 hours. In addition, Schnider et. al. [20] estimated a Cl_ss _of 1.89 liters/min, significantly larger than the experimental estimates of Campbell et. al. [48] and Morgan et. al. [49] of 1.02 to 1.6 liters [48,49].

In contrast, a much better estimate of V_ss _and Cl_ss _is obtained using the PBPK model. For the standard human (20% fat) the V_ss _for the PBPK model is about 1500 liters, of which about 97% is contributed by the adipose tissue. For body fat varying from 12% to 40%, the PBPK model V_ss _varies from 980 to 3000 liters, in good agreement with the experimental measurements of Campbell et. al. [48] and Morgan et. al. [49]. The intrinsic clearance, Tclr, (eq. (8)), varies from about 70 to 500 liters/min with a median value of about 160 liters/min (Table 4). This corresponds to a median value for the steady state liver clearance of about 1.49 liters/min/70 kg, with a range of 1 to 1.8 liters/min (Table 2), which, again, is similar to what is observed experimentally [48,49].

**Figure 12:**
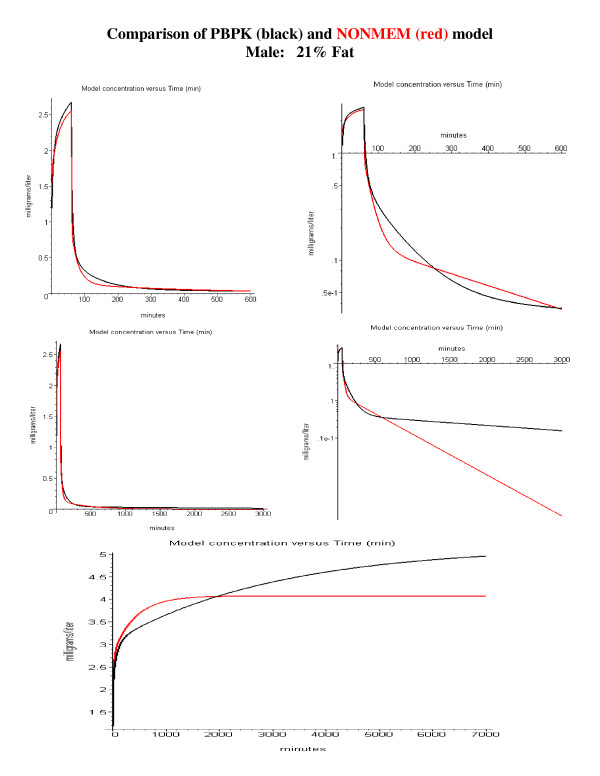
Comparison of NONMEM (Schnider et. al., Table 2 [20]) and PBPK model predictions for a standard male (21% body fat, age 53, weight 77 kg and height 177 cm). The top row shows the model predictions for a 1-hour constant infusion of 100 μg/kg/min followed by a 9 hour washout (left, absolute plot; right, semi-log plot). The middle row shows the model predictions when the washout period is extended to 49 hours. The bottom row compares the model predictions for a 5 day constant infusion of 100 μg/kg/min.

**Figure 13:**
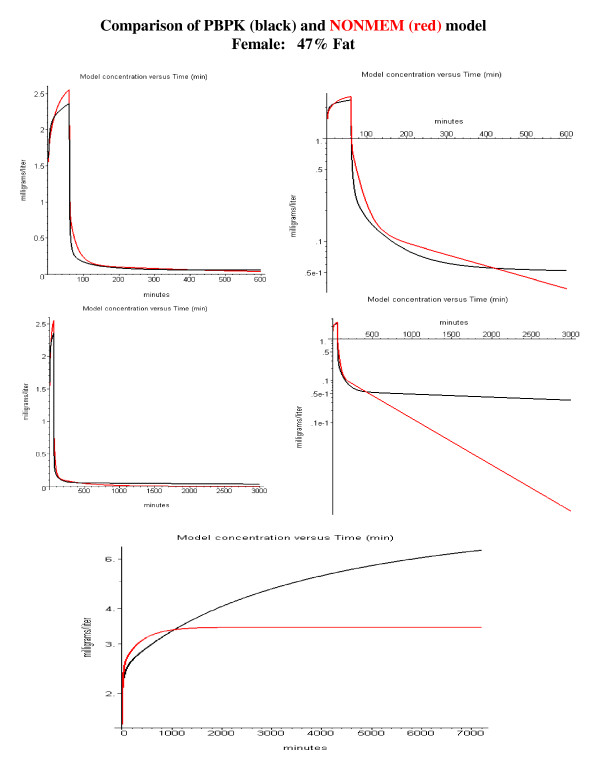
Same as fig. 12, except for an obese female with 47% body fat (age 53, weight 77 kg and height 150 cm).

A direct comparison of the PBPK and NONMEM model predictions is shown in fig. 12 (standard male, 21% body fat) and fig 13 (obese female, 47% body fat). The PBPK model used the average Tclr value for the young subjects (Tclr = 165) with the body fat determined using the subjects age, weight and height (eq. (2)). The top row compares the model predictions for a 1-hour constant infusion followed by a 9-hour washout. Both models give very similar results over this limited washout time. This is expected since this is the time period for which the NONMEM and PBPK parameters were optimized. The differences in the two models become dramatic if the washout is extended to 49 hours (fig. 12 and 13, middle row), long enough for differences in the value of V_ss _to become important. This underestimate of V_ss _has important clinical implications, which are illustrated in the bottom row of figs. 12 and 13 for the case of a constant 5-day infusion, mimicking the use of propofol for long term sedation. As can be seen in fig. 12, for the standard male (21% fat) the predicted steady state plasma value using the NONMEM model is about 19% less than that for the PBPK model using the "correct" value of V_ss_. This difference becomes significantly greater (53%) in obese subjects (fig. 13) with a larger adipose compartment.

### Physiological implications – body fat fraction

The major advantage of an accurate PBPK model is that it can be used to predict the pharmacokinetics for varying physiological conditions. The primary application of the PBPK approach has been in the field of toxicokinetics where the ability to predict the kinetics under varying physiological conditions is critical (for a recent review, see [51]). The usefulness of these PBPK predictions is critically dependent on having confidence that the PBPK model is not just a set of adjustable parameters but, instead, represents a true "physiologically based model" that can be extrapolated to conditions that are different from those that were used to derive the model parameters. The fact that this propofol PBPK model has just one new adjustable parameter (liver clearance), helps to justify this confidence.

**Figure 14:**
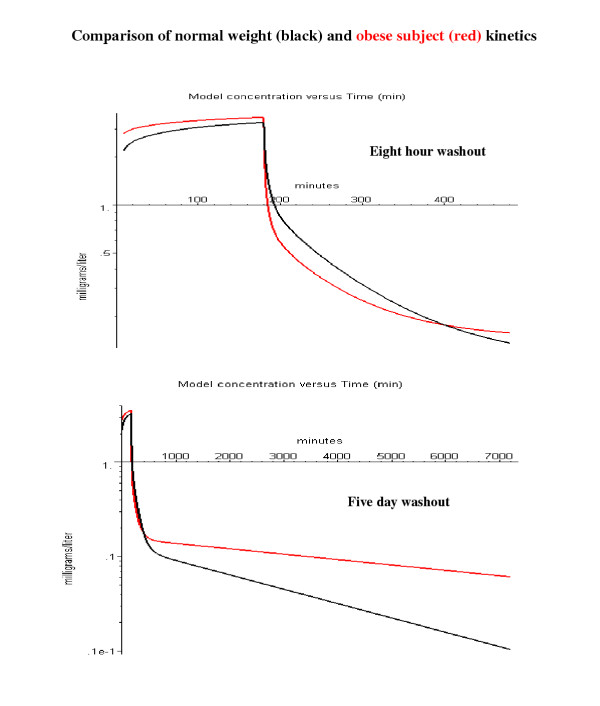
Comparison of arterial blood concentration in normal weight (black) and morbidly obese subjects (red). The propofol was infused at a constant rate of 0.1 mg/kg/min for 180 minutes and the washout was followed for 8 hours (top panel) or 5 days (bottom panel).

This section will focus on one physiological variable – the body fat fraction. The property that most distinguishes the propofol kinetics is its very high oil/water partition coefficient of about 4715. The corresponding adipose/blood partition coefficient is about 84 (eq. (4) which results in a huge volume of distribution, about 2000 liters/70 kg, of which about 97% is in the adipose tissue. One might expect that the body fat content should have a large influence on the kinetics. However, Servin et. al. [40] compared the propofol kinetics for normal weight and morbidly obese subjects during standard surgical anesthesia (180 minute constant infusion of 0.1 mg/kg/min followed by an 8 hour washout) and showed that the differences in the two groups of subjects was small. Figure 14 shows a PBPK model simulation of the experiments of Servin et. al. (see Methods for details). The black line is for a normal subject (20% body fat) and the red is for the average morbidly obese subject (50% body fat). Servin et. al. followed the washout for 8 hours after stopping the infusion (fig. 14, top panel). It can be seen that over most of the infusion and washout period, the concentrations in the obese subjects differs by less than 30% from that for the normal subjects, which is small compared to the inter-individual variation in the experimental study of Servin et. al. Thus, this model simulation is consistent with the main conclusion of Servin et. al. that variations in body fat content have relatively small effects on the propofol kinetics during standard surgical procedures. However, if the washout is followed for 5 days, a period that is long compared to the 33 hour adipose time constant, the difference in kinetics for the obese subjects becomes dramatic (fig. 14, bottom panel).

**Figure 15:**
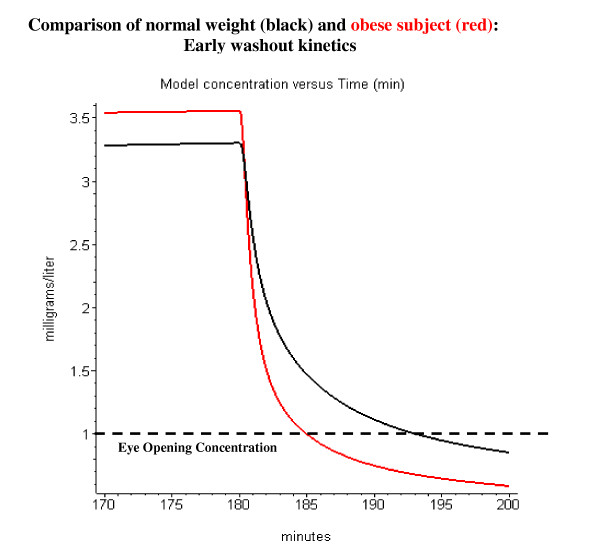
Comparison of time for eye opening after a 180 minute constant infusion in normal weight (black) and morbidly obese subjects (red). Same conditions as fig. 14, but only the first 20 minutes of the washout is plotted. The dashed line indicates the arterial concentration associated with the first eye opening.

The most significant difference that was observed by Servin et. al. [40] for the obese subjects is that they woke up significantly faster than the normal weight subjects after stopping the propofol infusion. Figure 15 shows the first 20 minutes of the washout period after the 180-minute propofol infusion. Servin et. al. recorded the time and arterial blood concentration of first eye opening. The eye opening arterial blood concentration was the same for obese and normal subjects (about 1 mg/l; fig. 15, dashed line). The model simulation in fig. 15 predicts an eye opening time of about 5 minutes for the obese subjects and 13 minutes for the normal subjects. This is qualitatively similar to what was observed by Servin et. al. (obese: 10.3 ± 6.3 minutes; normal: 18.4 ± 5.7 minutes).

**Figure 16:**
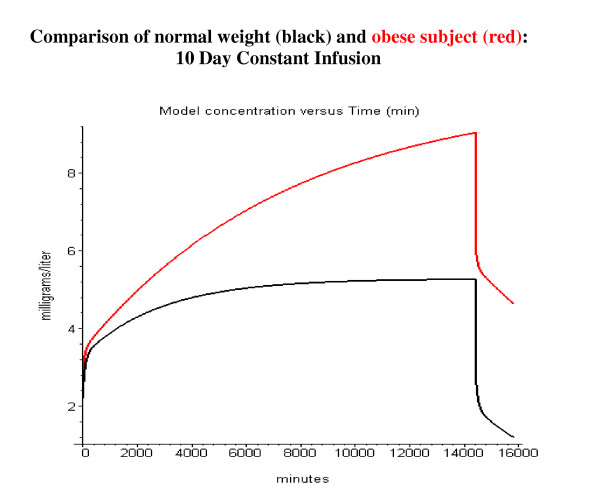
Comparison of arterial blood concentration in normal weight (black) and morbidly obese subjects (red). Ten-day constant infusion of 0.1 mg/kg/min.

**Figure 17:**
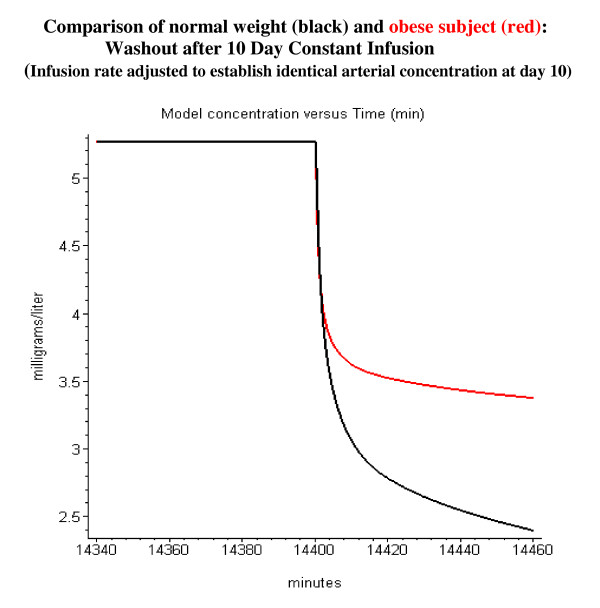
Comparison of washout kinetics in normal weight (black) and morbidly obese subjects (red). The 10 day constant infusion rate has been adjusted so that the concentration at the end of 10 days is identical for the normal and obese subjects (normal: 0.1 mg/kg/min; obese: 0.058 mg/kg/min). Only the first 60 minutes of the washout is plotted.

Propofol is routinely used for long-term sedation. Figure 16 shows the model simulation of the arterial concentration for a 10-day constant infusion of 0.1 mg/kg/min in normal weight and morbidly obese subjects. It can be seen that for these long-term infusions, the pharmacokinetics in obese subjects differs significantly from normal subjects. In normal weight subjects the arterial concentration reaches a steady state value after about 3 days, while in the obese subjects the arterial concentration is still rising at the end of the 10 day infusion. The arterial propofol rises to a higher concentration in the obese subjects because the same dose/kg was given to both subjects, while the obese subject has a lower rate of liver metabolism/kg because of the assumption of a constant liver weight per lean body mass. Probably the most important clinical implication of this model analysis is the prediction the kinetics of the washout phase after long-term sedation. Figure 17 shows the model simulation of the first 60 minutes of washout after a 10 day constant infusion. The infusion rate has been adjusted so that the normal and obese subjects have identical arterial concentrations just before the infusion is stopped. Sixty minutes after stopping the infusion the arterial concentration in obese subjects has dropped to a value of only 65% of the 10-day value, versus 45% in normal subjects. This suggests that blood concentrations during long-term infusions in obese subjects must be carefully monitored to assure rapid awakening when the infusion is terminated.

## Competing interests

The author(s) declare that they have no competing interests.

## Authors' contributions

D. G. L. performed the PBPK model development; the fitting of the PBPK parameters; and the analysis of the results.

T. W. S provided the experimental data and critically evaluated and significantly revised the manuscript.

## Pre-publication history

The pre-publication history for this paper can be accessed here:



## Supplementary Material

Additional File 1 A) A sample Maple worksheet for one subject. This worksheet can be used with the freely available PKQuest program to generate all the data and figures that were used for this subject in this paper. B) A detailed analysis and description of the use of the pharmacokinetics of the volatile anesthetics to determine the lipid fraction in different tissues. Click here for file
