# Supplementary material for: Human physiologically based pharmacokinetic model for propofol
Source: BMC Anesthesiol. 2005 Apr 22;5:4. doi: 10.1186/1471-2253-5-4 (PMC1090550; doi:10.1186/1471-2253-5-4)
Supplement: Additional File 1 — A) A sample Maple worksheet for one subject. This worksheet can be used with the freely available PKQuest program to generate all the data and figures that were used for this subject in this paper. B) A detailed analysis and description of the use of the pharmacokinetics of the volatile anesthetics to determine the lipid fraction in different tissues. [file 1471-2253-5-4-S1.doc]

**I. Sample Maple worksheet for propofol pharmacokinetics.**

> with(gensolve);

Data for Subject 102 - Average of 2 visits

propofol_102ave:=proc()

> local i,datafile,halftime,frdose,X,rate2,frdose2,rblpl1::name,klip1::name,age,sex,height,weight,inf_rate,rblplst,freeplst;

> defaultpar():

> Wtot:= 70; #Arbitrary - since dose/kg

standardhuman2(Wtot);

Age, weight, heigth and sex for subject 102 - find body fat

> age:=30;

sex:=1; #male

height:=1.7;

weight:=64.4;

> Fat:=bodyfat(height,weight,age,sex)/100;

freepl = fraction free in plasma water

> freepl:=0.022;

Tclr[liver] = free water liver clearance

> Tclr[liver]:=478;

Pulmonary sequestration: 40% sequestered and released with 200 minute halftime.

> frdose:=0.6;

halftime:=200;

3 different propofol input: 1) 20 second bolus equal to bolus fraction non-sequestered; 2) Exponential input = sequestered bolus fraction and 3) the 1 hour constant infusion starting at 60 minutes

> ninput :=3;

> X:=20.0/60; #20 second bolus injection of 2 mg/Kg

finput[1]:=table([organ=vein,type=1,rate=2*frdose*Wtot/X,tbeg=0,tend=X,csteady=0, padjust=0]);

finput[2]:=table([organ=vein,type=2,rate=2*Wtot*(1-frdose)/halftime,tbeg=0, tend=halftime,csteady=0,padjust=0]);

inf_rate:=0.050;#constant infusion rate in mg/Kg/min

rate2:=inf_rate*Wtot;#Constant 60 min infusion of 25 ug/kg/min starting at 60 min.

finput[3]:=table([organ=vein,type=1,rate=rate2,tbeg=60, tend=120,csteady=0,padjust=0]);

cunit:="milligrams";

> concunit[vein] :=4;#plasma concentration of vein

findabs:=0;

freeplst:=0.02;rblplst:=1.0;

> Kfwat:=4715;

Call procedure that calculates blood plasma ratio (rblpl) and fraction lipid in blood (klip1)

> free_eqs_correct(Kfwat,rblplst,freeplst,wfractpl,wfract[vein],freepl,rblpl1,klip1);

rblpl:=rblpl1;klip[vein]:=klip1;

De[liver]:=0.3;#Dispersion parameter for liver

pdata:=1;

Data for subject 102 - averaged for both visits

> data[1]:=[[2.1, 2.670000000], [4., 1.190000000], [8.01, .6290000000], [16., .4635000000], [30.03, .1900000000], [59.72, .1515000000], [62.03, .7660000000], [64.01, .9585000000], [68.01, 1.065000000], [76.02, 1.090500000], [90., 1.185000000], [119.5, 1.200000000], [122.01, .5400000000], [124., .4555000000], [128., .3555000000], [136., .3060000000], [150., .1985000000], [180.03, .1740000000], [240., .9555000000e-1], [300., .6795000000e-1], [599.25, .3500000000e-1]];

> print("data[1]=",data[1]);

> end proc:

>

> free_eqs_correct:=proc(Kfwat,rblplst,freeplst,wfractpl,wfractbl,freepl,rblpl1::name,klip1::name)

local freec,rblpl2,klip2;

freec:=hmt*freeplst/(rblplst-1+hmt);

rblpl2:=hmt*freepl/freec+1-hmt;

klip2:=wfractpl*rblpl2/(freepl*Kfwat) - wfractbl/Kfwat;

rblpl1:=rblpl2;klip1:=klip2;

printf("Using freepl=%5.4f to determine rblpl=%5.4f and klip[vein]=%5.4f\n",freepl,rblpl2,klip2);

end proc:

**II. Use of volatile anesthetics to determine the lipid fraction in different tissues.**

The partition of the volatile anesthetics in human tissue can be described using a very simple mechanistic basis. Their partition can be well described assuming that they partition in the tissue water and lipid on the basis of their of their oil/water partition coefficient. Using this assumption, one can derive the relationship between the oil/air (K_oa_) and water/air (K_wa_) partition coefficients and the fraction of lipid in the different organs.

The total concentration of solute in the tissue (C_T_) is equal to:

$$C_{T}=v_{w}C_{w}+v_{f}C_{f}$$

where v_w_ and v_f_ are the fraction of water and fat in the tissue and c_w_ is the free water concentration and c_f_ is the fat concentration. The tissue/water partition is then:

$$C_{T}/C_{w}=v_{w}+v_{f}C_{f}/C_{w}$$

Solving this equation for v_f_ (the tissue fat fraction):

$$v_{f}=(K_{tw}-v_{w})K_{wa}/K_{oa}$$

where the following definitions have been used:

$$\begin{matrix} tissue/water partition=K_{tw}=C_{T}/C_{w} \\ water/air partition= K_{wa}=C_{w}/C_{air} \\ oil/air partition = K_{oa}=C_{f}/C_{air} \\ oil/water partition=K_{oil}=K_{oa}/K_{wa} \end{matrix}$$

The tissue fat fraction (v_f_) of the different organs in the PBPK model was estimated by application of eq. to experimental data for the volatile anesthetics. This parameter is an empirical value that results in the correct experimental tissue partition coefficient. The following Table lists the results of this calculation for four different anesthetics. The values of the tissue/air partition coefficient in this table were determined by equilibrating homogenized human autopsy tissue with the different anesthetics at 37 °C. It can be seen that there is a similar set of values of v_f_ over a wide range of values of oil/air and water/air partition coefficients using data from three different publications.

| Solute | Reference | water/air | oil/air | Tissue | tiss/blood | tiss/air | tiss/wat | wat_fract | fat_fraction |
| --- | --- | --- | --- | --- | --- | --- | --- | --- | --- |
|  |  |  |  |  |  |  |  |  |  |
| isoflurane | [1] | 0.61 | 90 | blood | 1 | 1.4 | 2.295082 | 0.82 | 0.009998 |
|  |  | 0.61 | 90 | brain | 1.74 | 2.436 | 3.993443 | 0.8 | 0.021644 |
|  |  | 0.61 | 90 | heart | 1.62 | 2.268 | 3.718033 | 0.8 | 0.019778 |
|  |  | 0.61 | 90 | liver | 2 | 2.8 | 4.590164 | 0.7 | 0.026367 |
|  |  | 0.61 | 90 | muscle | 1.52 | 2.128 | 3.488525 | 0.78 | 0.018358 |
|  |  | 0.61 | 90 | adipose | 53.1 | 74.34 | 121.8689 | 0.2 | 0.824644 |
|  |  |  |  |  |  |  |  |  |  |
| methoxyflurane | [1] | 4.33 | 970 | blood | 1 | 14.5 | 3.34873 | 0.82 | 0.011288 |
|  |  | 4.33 | 970 | brain | 1.26 | 18.27 | 4.2194 | 0.8 | 0.015264 |
|  |  | 4.33 | 970 | heart | 1.07 | 15.515 | 3.583141 | 0.8 | 0.012424 |
|  |  | 4.33 | 970 | liver | 1.53 | 22.185 | 5.123557 | 0.7 | 0.019746 |
|  |  | 4.33 | 970 | muscle | 1.54 | 22.33 | 5.157044 | 0.78 | 0.019539 |
|  |  | 4.33 | 970 | adipose | 52.47 | 760.815 | 175.7079 | 0.2 | 0.783453 |
|  |  |  |  |  |  |  |  |  |  |
| Desflurane | [2] | 0.225 | 18.7 | blood | 1 | 0.424 | 1.884444 | 0.82 | 0.012807 |
|  |  | 0.225 | 18.7 | brain |  | 0.62 | 2.755556 | 0.8 | 0.023529 |
|  |  | 0.225 | 18.7 | heart |  | 0.57 | 2.533333 | 0.8 | 0.020856 |
|  |  | 0.225 | 18.7 | liver |  | 0.87 | 3.866667 | 0.7 | 0.038102 |
|  |  | 0.225 | 18.7 | muscle |  | 0.62 | 2.755556 | 0.78 | 0.02377 |
|  |  | 0.225 | 18.7 | adipose |  | 15.3 | 68 | 0.2 | 0.815775 |
|  |  |  |  |  |  |  |  |  |  |
| Halothane | [2] | 0.8 | 220 | blood | 1 | 2.32 | 2.9 | 0.82 | 0.007564 |
|  |  | 0.8 | 220 | brain |  | 3.43 | 4.2875 | 0.8 | 0.012682 |
|  |  | 0.8 | 220 | heart |  | 3.61 | 4.5125 | 0.8 | 0.0135 |
|  |  | 0.8 | 220 | liver |  | 5.23 | 6.5375 | 0.7 | 0.021227 |
|  |  | 0.8 | 220 | muscle |  | 3.76 | 4.7 | 0.78 | 0.014255 |
|  |  | 0.8 | 220 | adipose |  | 138 | 172.5 | 0.2 | 0.626545 |
|  |  |  |  |  |  |  |  |  |  |
| Desfluane | [3] | 0.225 | 18.7 | blood | 1 | 0.424 | 1.884444 | 0.82 | 0.012807 |
|  |  | 0.225 | 18.7 | brain |  | 0.54 | 2.4 | 0.8 | 0.019251 |
|  |  | 0.225 | 18.7 | heart |  | 0.54 | 2.4 | 0.8 | 0.019251 |
|  |  | 0.225 | 18.7 | liver |  | 0.55 | 2.444444 | 0.7 | 0.020989 |
|  |  | 0.225 | 18.7 | muscle |  | 0.94 | 4.177778 | 0.78 | 0.040882 |
|  |  | 0.225 | 18.7 | adipose |  | 12 | 53.33333 | 0.2 | 0.639305 |
|  |  | 0.225 | 18.7 | kidney |  | 0.4 | 1.777778 | 0.2 | 0.018984 |
|  |  |  |  |  |  |  |  |  |  |
| Halothane | [3] | 0.8 | 220 | blood | 1 | 2.32 | 2.9 | 0.82 | 0.007564 |
|  |  | 0.8 | 220 | brain |  | 4.79 | 5.9875 | 0.8 | 0.018864 |
|  |  | 0.8 | 220 | heart |  | 4.6 | 5.75 | 0.8 | 0.018 |
|  |  | 0.8 | 220 | liver |  | 5.13 | 6.4125 | 0.7 | 0.020773 |
|  |  | 0.8 | 220 | muscle |  | 9.5 | 11.875 | 0.78 | 0.040345 |
|  |  | 0.8 | 220 | adipose |  | 136 | 170 | 0.2 | 0.617455 |
|  |  | 0.8 | 220 | kidney |  | 2.85 | 3.5625 | 0.2 | 0.012227 |

References:

1. Lerman J, Schmitt-Bantel BI, Gregory GA, Willis MM, Eger EI, 2nd: **Effect of age on the solubility of volatile anesthetics in human tissues**. *Anesthesiology* 1986, **65**(3):307-311.

2. Zhou JX, Liu J: **The effect of temperature on solubility of volatile anesthetics in human tissues**. *Anesth Analg* 2001, **93**(1):234-238.

3. Yasuda N, Targ AG, Eger EI, 2nd: **Solubility of I-653, sevoflurane, isoflurane, and halothane in human tissues**. *Anesth Analg* 1989, **69**(3):370-373.
